# Canine influenza virus coinfection with *Staphylococcus pseudintermedius* enhances bacterial colonization, virus load and clinical presentation in mice

**DOI:** 10.1186/s12917-016-0708-6

**Published:** 2016-06-04

**Authors:** Dildar Hussain Kalhoro, Shanshan Gao, Xing Xie, Shan Liang, Su Luo, Yanbing Zhao, Yongjie Liu

**Affiliations:** College of Veterinary Medicine, Nanjing Agricultural University, Nanjing, 210095 China

**Keywords:** canine influenza virus, *Staphylococcus pseudintermedius*, coinfection, pathological lesions, mice

## Abstract

**Background:**

Canine influenza virus (CIV) and *Staphylococcus pseudintermedius* (Sp) are pathogens that cause respiratory disease in dogs. Considering bacterial infections following influenza are a leading cause of illness and death, it is of particular meaning to investigate the interaction between these two pathogens. In this study, BALB/c mice were used as a mouse model to assess whether inoculation with CIV H3N2 followed by *S. pseudintermedius* 72 h later resulted in exacerbation of disease. Disease was characterized by assessment of body weight loss, titration of virus and bacteria, histopathology, and cytokine production*.*

**Results:**

There was a significantly greater decrease in body weight in the co-infected group compared with the CIV-only and SP-only groups. CIV inoculation increased bacterial colonization, whereas secondary infection with *S. pseudintermedius* elevated the viral RNA load of CIV in tissues. The histological lesions in the brain, spleen and lung were more severe in the CIV/Sp group than in the singly treated groups. Infection with CIV alone, Sp alone or coinfection stimulated a significantly higher release of cytokines, such as interferon-gamma (IFN)-γ, interleukin 6 (IL)-6, tumor necrosis factor (TNF-α) and lymphotactin (Lptn), than was observed in the mock-infected group (PBS). Moreover, the levels of IFN-γ in the spleen and lung were higher in the CIV/Sp group compared with the CIV-only and Sp-only groups.

**Conclusion:**

Our findings provide the first demonstration that the secondary infection of mice with Sp leads to increased clinical signs and lesions during canine influenza.

**Electronic supplementary material:**

The online version of this article (doi:10.1186/s12917-016-0708-6) contains supplementary material, which is available to authorized users.

## Background

Canine infectious respiratory disease (CIRD) is a common, worldwide disease syndrome, and involves a number of viral and bacterial pathogens. Canine influenza virus (CIV) is one of the viral causes of contagious respiratory disease [1]. In January 2004, CIV subtype H3N8 was first reported in racing greyhound dogs in Florida [2]. Subsequently, in 2007, a different influenza virus, subtype H3N2, caused an outbreak of canine respiratory disease in South Korea [3]. Recent reports suggest that H3N2 CIV has become endemic in the canine populations in China [4–6].

Bacterial pathogens associated with CIRD include *Bordetella bronchiseptica*, *Streptococcus equi* subspecies *zooepidemicus* and *Mycoplasma* [7]. In humans, Staphylococcal species are frequently identified as the cause of bacterial pneumonia [8, 9]. A study from our group showed that *Staphylococcus pseudintermedius* (Sp) was the most frequently isolated bacterial pathogen (24/40) from respiratory cases (unpublished data). This bacterium has been previously misidentified as *S. intermedius*. The name *S. pseudintermedius* is now given to the canine-specific strain of *S. intermedius* [10]. *S. pseudintermedius* is a common commensal of oral and nasal vacities and the skin in healthy dogs, where it can also cause invasive disease [11, 12]. Sasaki et al. [13] isolated Sp from inpatient dogs (46.2 %) and outpatient dogs (19.4 %) in a Japanese veterinary teaching hospital. Some recent reports confirmed this bacterium can spread from pets to their owners [14, 15] and to the veterinary staff working in the veterinary clinics [16]. Furthermore, methicillin resistant *S. pseudintermedius* (MRSP) has emerged as an increasingly important cause of opportunistic infections in dogs [17]. It may emerge as a major and difficult-to-treat pathogen in the future.

In humans, co-infections of influenza virus and respiratory bacterial pathogens are common, and bacterial secondary infections have played an important role in influenza virus pandemics [18, 19]. Influenza pneumonia occurs mainly due to virus-bacteria interactions because of a synergistic effect that occurs during invasion of the respiratory tract [20]. Epithelial and mucosal degradation occurs due to viral replication, resulting in a reduced innate immune response to prevent secondary bacterial infections [21]. Thus, bacterial infections may be the main cause of death related to influenza virus infection in the absence of a pre-existing co-morbidity [22, 23].

Although CIV infection is typically self-limited with high-morbidity but low mortality, co-infections with respiratory bacterial pathogens may impact the pathogenicity and course of disease. It will be of particular importance considering the role of secondary bacterial infections as a major complication of influenza in pet clinics. To understand the treatment procedures for fighting viral and secondary bacterial infections, it is important to study the mechanisms involved in the interaction between influenza virus and bacterial organisms. In the present study, we investigated the interactions between CIV and Sp and evaluated pathological lesions in mice.

## Methods

### Infectious agents

The viral strain A/Canine/Jiangsu/06/2010 (H3N2) was used in this study. The viral strain was isolated from nasopharyngeal swabs of dogs with severe respiratory syndrome, which were collected from Animal Clinics of Nanjing Agricultural University in Jiangsu Province of China in 2010. The GenBank accession numbers are JN247615 to JN247623. Plaque assays were performed on monolayers of Madin-Darby canine kidney (MDCK) cells in 12-well tissue culture plates to determine the viral titres [24]. Viral aliquots were diluted in phosphate-buffered saline (PBS) supplemented with 0.1 % bovine serum albumin and adjusted to a final concentration of 1x10^5^ plaque forming units (PFU)/ml [25]. The Sp NJ-1 strain was isolated from a dog with respiratory syndrome. The bacterial species was identified based on its biochemical characteristics [26] and sequencing of the 16S rRNA gene. To prepare the inoculum, the bacterial strain was cultured in Luria–Bertani (LB) broth at 37 °C for 4 h. A suspension with approximately 1x10^9^ colony forming units (CFU)/ml was made in phosphate-buffered saline (PBS).

### Mice

BALB/c mice (6 weeks of age, 18–20 g) were purchased from the Animal Experiment Centre, Yangzhou University, China. All of the mice were housed in individual compartments in stainless-steel wire cages. Before conducting the study, approval for conducting the experiments was obtained from the Animal Ethics Committee of Nanjing Agricultural University.

### Animal experiments

One hundred mice were randomly divided into four groups with 25 mice per group. The mice in group 1 (Sp-only) were intranasally inoculated with Sp(1×10^9^ CFU/ml, 50 μl/mouse) and 72 h later inoculated with non-infectious MDCK cell culture supernatant (50 μl/mouse); the mice in group 2 (CIV-only) were intranasally inoculated with PBS (50 μl/mouse) and 72 h later inoculated with CIV (1×10^5^ PFU/ml, 50 μl/mouse); the mice in group 3 (CIV/Sp) were intranasally inoculated with CIV and 72 h later inoculated with Sp; and the mice in group 4 were inoculated with non-infectious MDCK cell culture supernatant and 72 h later inoculated with PBS. The groups were housed in individual isolation rooms.

The daily weight and clinical signs were recorded for up to five days. The observers were blinded as to the experimental treatments and they had veterinary medical qualifications to make assessments about clinical signs. Three mice from each group were killed humanely according to a pre-designated schedule at indicated time points. Blood samples from the experimental groups were collected using sodium heparin tubes from the eyeball capillary before collecting tissues, including spleen, lung and brain, at 1, 2, 3, 4 and 5 days post-infection (d.p.i.).

### Pathological analysis

The lung, spleen and brain tissues from mice belonging to the various groups were harvested at 2 days post Sp challenge in the co-infected group and were fixed in 10 % formalin. After fixation, the tissues were embedded in paraffin wax. Tissue sections with a thickness of 4 μm were stained with haematoxylin and eosin (H&E) and assessed for the degree of inflammation and necrosis. The lungs were assigned a grade 0 to 3 based on the histological characteristics as described by Alymova et al. [27]. A score of 0 was given when no pathological changes could be detected. A score of 1 was given to findings representing mild pathologies, including minimal infiltrates of lymphocytes and plasma cells around airways and vessels, minimal epithelial hyperplasia, minimal leukocyte infiltration of alveolar spaces, and <10 % of the lung affected. A score of 2 was given for findings representing moderate pathologies, including moderate infiltrates of lymphocytes and plasma cells around airways and vessels, moderate epithelial hyperplasia with focal necrosis, focally extensive infiltration of the alveolar spaces by leukocytes with some consolidation, focal pleuritis, and >10 % but <30 % of the lung affected. A score of 3 was given for findings representing pathologies of greater severity, including extensive necrosis of airway epithelium and of interstitium, extensive leukocyte infiltration and consolidation, severe pleuritis, and lobar involvement. Grading and descriptions of pathology were performed by an experienced veterinary pathologist in a single-blinded manner.

### Bacterial isolation

Samples of lung, spleen and brain from each mouse were weighed and homogenized individually in PBS to obtain a 10 % weight-to-volume suspension. The number of CFUs of Sp was determined by plating serial 10-fold dilutions of homogenates or blood on LB agar in duplicate. Therefore, the results are reported as CFU/ml for blood and as CFU/g for the lung, spleen and brain.

### Determination of viral loads

The tissues were homogenized in lysates at a ratio of 1:1 (g/ml) and centrifuged at 10,000 *g* for 30 min, and the supernatants were collected for viral RNA extraction using a Virus Nucleic Acid Extraction Kit II (Geneaid, Taiwan). Quantitative RT-PCR (RT-qPCR) was performed to measure the viral loads in the above-mentioned organs and blood samples. The RNA from the samples was reverse-transcribed and run in an ABI 7300 Real-time PCR System using a SYBR1 Premix Ex Taq™ (Perfect Real Time) kit (TaKaRa, Dalian). The following primers were designed and used on the matrix gene region: 5′-TCTATCGTCCCATCAGGC/GGTCTTGTCTTTAGCCATTC-3′. Simple Vector pMD19-T (50 ng/μl; TaKaRa, Dalian) containing the target virus sequence was used as a reference standard. Series of eight 10-fold dilutions equivalent to 1×10^3^-1×10^10^ copies per reaction were prepared to generate calibration curves and were run in parallel with the test samples [28].

### Quantification of cytokine levels

The cytokine level was assessed to determine the correlation between severe disease and inflammatory cytokine production in the mice. The spleen and lung from the mice in all of the groups were homogenized in 1 ml of PBS/1 g of tissues. The homogenates were centrifuged, and the supernatants were frozen at −70 °C until testing. ELISAs for the levels of interferon-gamma (IFN-γ), interleukin 6 (IL-6), lymphotactin (Lptn) and tumor necrosis factor (TNF-α) on the lung and spleen from infected mice were performed using ELISA kits (Sigma-Aldrich, Beijing) according to the manufacturer’s instructions.

### Statistical analysis

The data were collected and analysed using MS Excel 2010 and SPSS Statistics v20.0 software. The weight loss/gain, bacterial load, viral load and cytokine levels were analysed by analysis of variance (ANOVA) followed by Turkey’s multiple comparison test [29, 30]. Differences with *P*<0.05 were considered statistically significant, and differences with *P*<0.01 were considered highly significant.

## Results

### Clinical signs during experimental infection in the mice

The mice showed normal features after bacterial inoculation, but after 18 h post-infection (h.p.i), all the infected mice manifested with lethargy, anorexia, huddling and ruffled fur. It is notable that nervous signs appeared in the mice of SP (2/25) and CIV/Sp (4/25) groups at 20 h.p.i. On 2 d.p.i., this phenomenon was more pronounced. Distinct central nervous disturbances, including bending of their head towards one side and walking in circles, were observed in the Sp and CIV/Sp groups. More severe clinical signs were observed in the CIV/Sp group when the mice were lifted by the tail. Such neurological signs lasted until the end of experiment. But the number of affected mice did not increase. Mice with neurological signs lost more body weight compared with the other mice in the same group. In contrast, the mice in the CIV group did not show any neurological signs, but the signs, such as depression, decreased activity, huddling and ruffled fur, were witnessed in all the infected mice from 24 h.p.i., and lasted until the end of experiment

### Body weight changes in mice

**Fig. 1:**
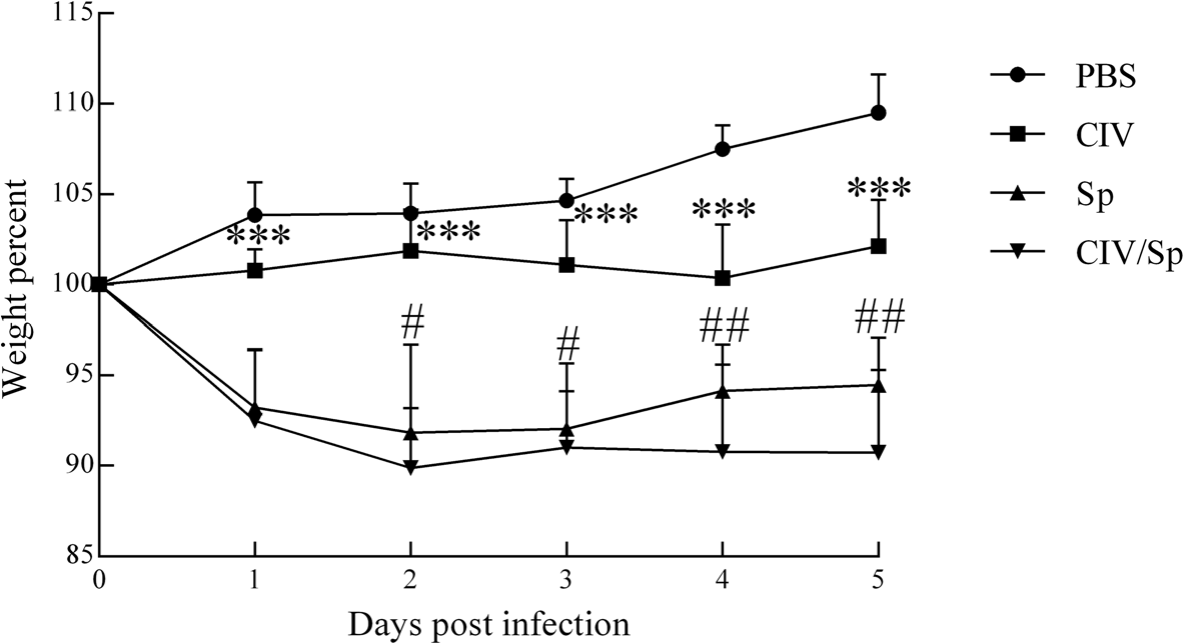
Changes in the body weights of BALB/c mice that were infected with CIV or *S. pseudintermedius* alone*,* coinfected with both, or exposed to PBS as a control. The percentage of the body weight was calculated relative to the body weights recorded five days post-challenge. Analysis of variance (ANOVA) was performed for the statistical analysis. ***, *P* < 0.001, indicates a significantly different weight percent for CIV groups compared with CIV/Sp groups. #, *P* < 0.05, ##, *P* < 0.01, or ###, *P* < 0.001, indicates a significantly different weight percent for Sp groups compared with CIV/Sp groups

All of the infected groups exhibited a lowered body weight compared with the PBS group (Fig. 1, Additional file 1). Mice inoculated with virus alone showed mild to modest disease severity. There was a significant difference (*P*<0.05) between the sham and CIV groups only at 4 and 5 d.p.i. In contrast, Sp-alone-inoculated mice showed signs of more severe illness, and there was a significant difference between the sham and Sp groups (*P*<0.001) at each time point. Significant differences were also observed between the sham and CIV/Sp groups (*P*<0.001) at each time point. Mice coinfected with CIV and Sp lost more body weight compared with the mice in both the CIV-only (*P*<0.001) and Sp-only (*P*<0.05 or *P*<0.01) groups at 2, 3, 4 and 5 d.p.i., indicating that mice coinfected with CIV followed by *S. pseudintermedius* developed more severe disease.

### Determination of viable bacteria and viral RNA loads in the tissues of infected mice

**Fig. 2:**
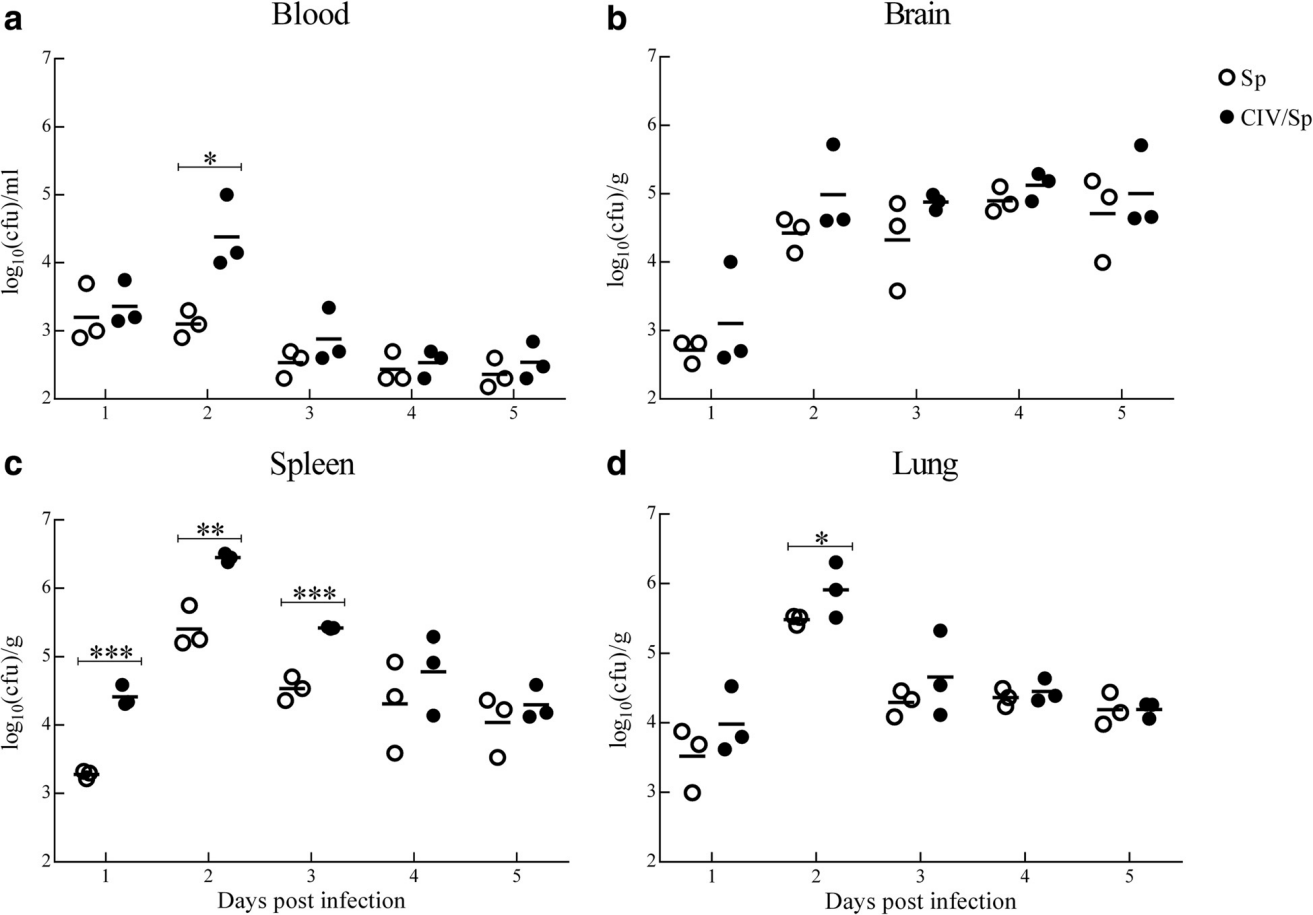
Dynamic change in the bacteria loads in the blood, brain, spleen and lung (**a**, **b**, **c** and **d**) of mice infected with *S. pseudintermedius* alone or both *S. pseudintermedius* and CIV. The bacterial loads in the infected animals were measured on days 1, 2, 3, 4 and 5 post-challenge. The results are expressed as the mean log10 cfu/ml or log10 cfu/g. Each data point represents a single mouse, and the bar indicates the average for the group. Analysis of variance (ANOVA) was performed for the statistical analysis. *, *P* < 0.05, **, *P* < 0.01, or ***, *P* < 0.001

The bacterial loads in the blood, brain, spleen and lungs of mice infected with bacteria alone and with both virus and bacteria were compared (Fig. 2, Additional file 1). At each time point, the bacterial load in all of the tissues of the coinfected group was relatively higher than that in the bacteria- alone group, but the statistically significant differences were not necessarily found in each tissue. At 1 d.p.i., the bacterial load in the blood, lung and brain was slightly higher in the CIV/SP group compared with that in the Sp-only group, but the difference was not significant (*P*>0.05). However, the bacterial loads in the spleen showed significantly higher level in the CIV/Sp group than in the Sp-only group (*P*<0.01). At 2 d.p.i., the bacterial loads in the blood, brain, spleen and lung had reached a peak level, and showed a significant higher level in the blood, spleen and lung of the coinfection group compared to the SP-lonely group (*P*<0.05 or *P* < 0.01). At 3 d.p.i., bacterial load was significantly higher in the spleen of CIV/Sp group than that in the SP-lonely group (*P*<0.01), and at 5 d.p.i., the bacterial loads in the blood, brain, spleen and lung were decreased to more than 10^2^, 10^5^, 10^4^ and 10^4^ CFU/g (CFU/ml) in both the two groups, respectively.

**Fig. 3:**
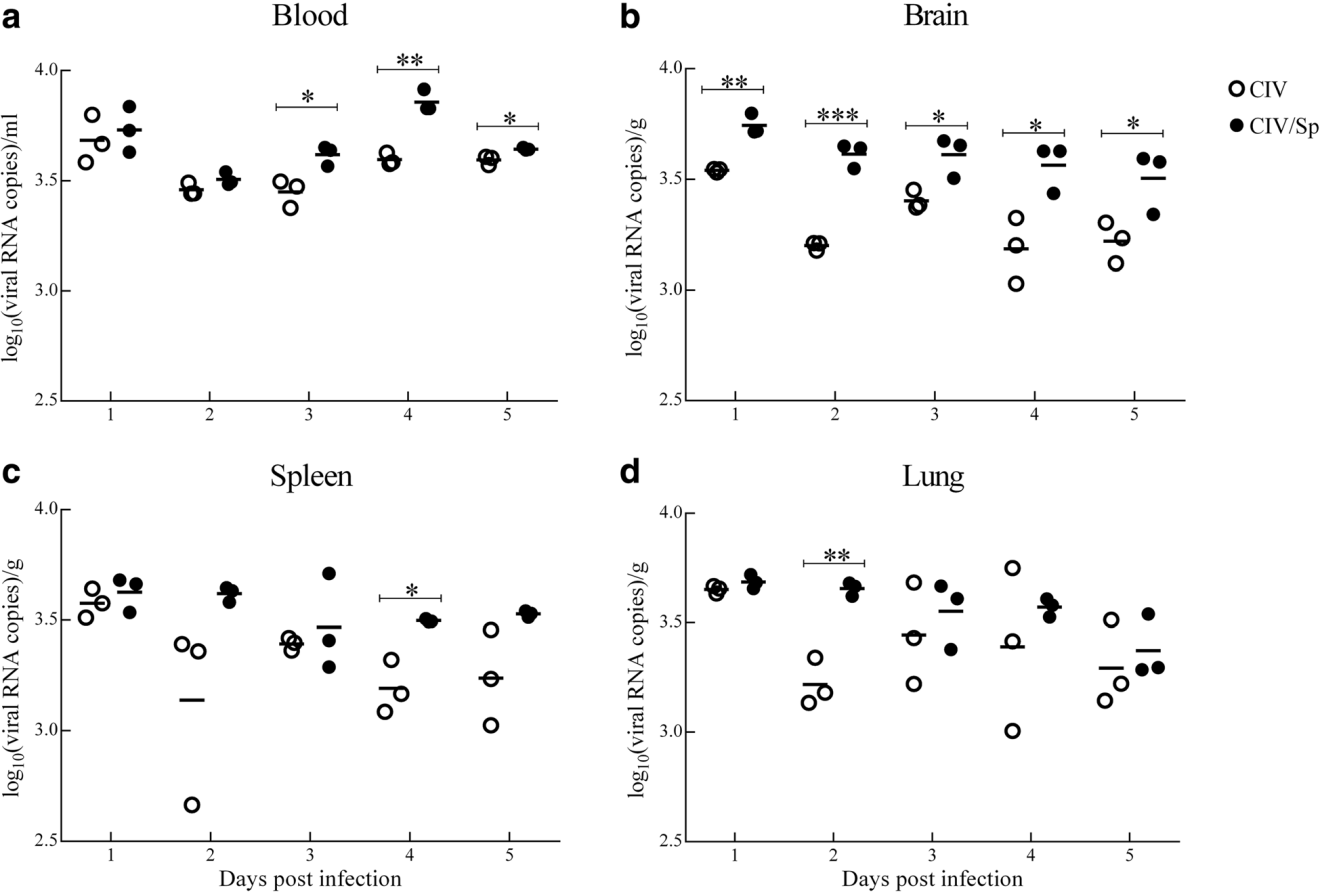
Dynamic change in the viral loads in the blood, brain, spleen and lung (**a**, **b**, **c** and **d**) of mice infected with CIV alone, or both *S. pseudintermedius* and CIV. The viral loads in the infected animals were measured on days 1, 2, 3, 4 and 5 post-challenge. The results are expressed as the log10 RNA copy numbers per gram or per millilitre of sample. Each data point represents a single mouse, and the bar represents the average for the group. Analysis of variance (ANOVA) was performed for the statistical analysis. *, *P* < 0.05, **, *P* < 0.01, or ***, *P* < 0.001

RT-qPCR was used to measure the viral loads in the above tissues of mice infected with virus alone and with both virus and bacteria (Fig. 3, Additional file 1). At all time-points that virus was detected at, the viral load in all the tissues of the coinfected group was higher than that in the virus-alone group. Statistically, compared to CIV-only group, the viral loads in the blood was significantly higher at 3, 4 and 5 d.p.i. in the CIV/Sp group. The brain showed more significant changes. At each time point, brain viral loads in the CIV/Sp group were higher. The viral loads in the CIV/Sp group showed significantly higher levels in the spleen at 4 d.p.i. and in the lung at 2 d.p.i than those in CIV-only group.

### Histopathology

**Fig. 4:**
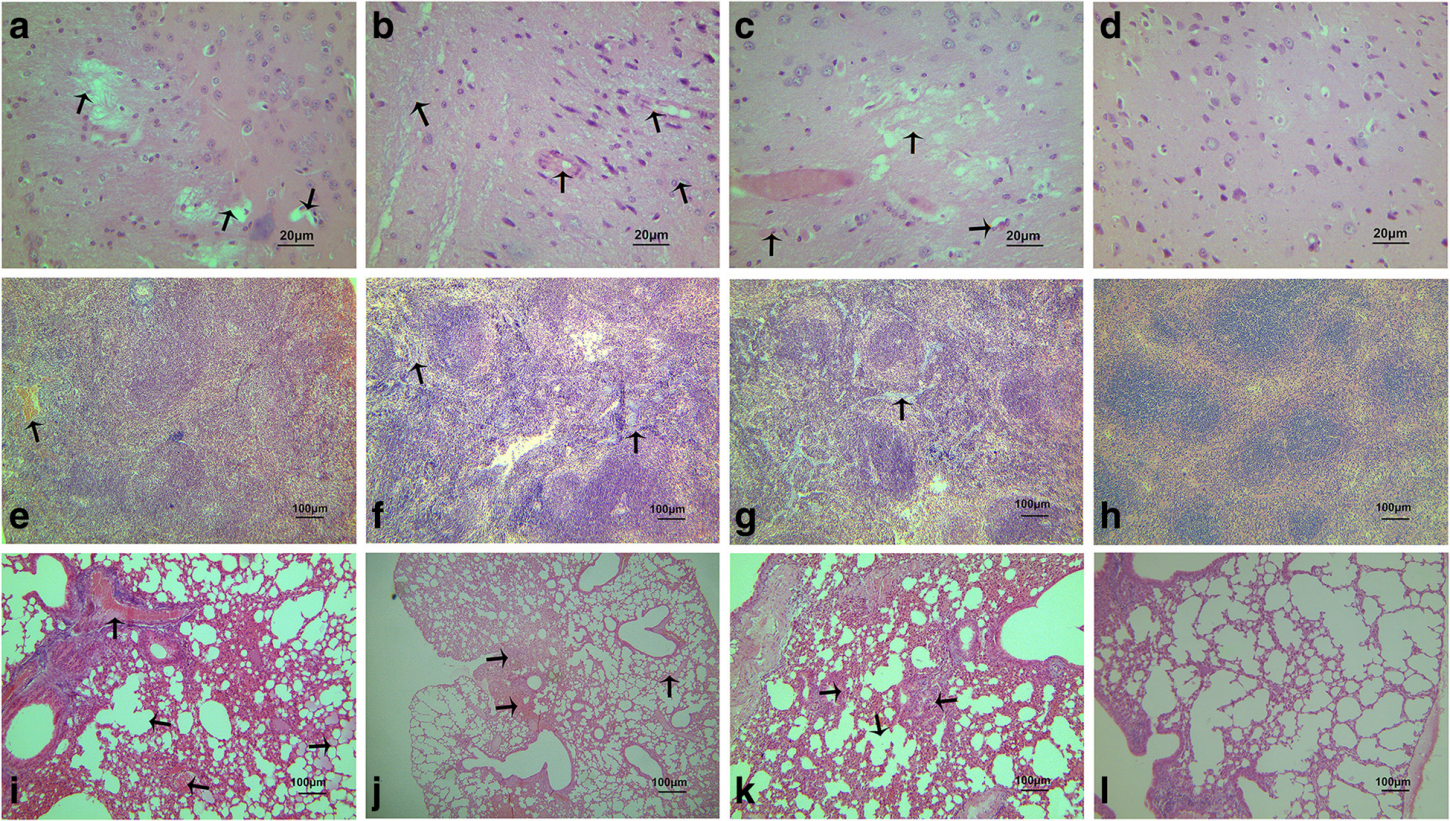
Histopathological lesions at 2 days post Sp challenge in the co-infected group, shown by H&E staining in the brain, spleen and lung of mice infected with CIV alone, *S. pseudintermedius* alone, or both. **a** Representative brain image from the CIV-only group, showing diffused hyperplasia of microglial cells and disintegration of neuron cells (400×). **b** Representative brain image from the Sp-only group, showing dissolution of nerve fibres, vascular cuff phenomena and neurophagia phenomena (400×). **c** Representative brain image from the CIV/Sp group, showing nerve fibre dissolution and disappearance of neurons (400×). **e** Representative spleen image from the CIV-only group, showing no lesions (100×). **f** Representative spleen image from the Sp-only group, showing hyperplasia of the splenic trabeculae (100×). **g** Representative spleen image from the CIV/Sp group, showing hyperplasia and degeneration of the splenic trabeculae (100×). **i** Representative lung image from the CIV-only group, showing widening of the pulmonary interstitial (100×). **j** Representative lung image from the Sp-only group, showing widening of the alveolar septa (100×). **k** Representative lung image from the CIV/Sp group, showing severe fibrin exudation, appearance of neutrophils and widening of the alveolar septa (100×). **d**, **h**, **l** Representative brain, spleen and lung images of the control group, showing no histopathological lesions (400×, 400× and 100×)

The extent of histological lesions in the brain, spleen and lungs of mice infected with CIV/Sp and with either Sp or CIV alone was compared at 2 d.p.i. All of the tissues tested in the Sp-only, CIV-only and CIV/Sp groups showed significant lesions, whereas the mice in the PBS group showed no lesions in the lungs, brain and spleen (Fig. 4).

The brain of the mice in the CIV-only group exhibited the dissolution of nerve fibres. In some areas, a diffused hyperplasia of microglial cells was noticed. In addition, the disintegration of neuronal cells was observed (Fig. 4a). The brain of the mice in the Sp-only group showed dissolution of a large area of nerve fibres. The microglial cells were severely diffused. The characterizing feature of the Sp group was the appearance of vascular cuff and neurophagia phenomena (Fig. 4b). Microscopic lesions in coinfected mice included changes seen in mice infected with single agents, but tended to be more severe than in mice infected with either agent alone. The nerve fibre dissolution observed in the CIV/Sp group was more obvious compared with that in the other two groups, and the disappearance of neurons was also observed (Fig. 4c). The spleen in the CIV-only group did not present any lesions (Fig. 4e). Histopathological lesions in spleens from Sp-infected mice were characterized by marked accumulations of macrophages, which expanded marginal zones, and there was hyperplasia of the lymphoid tissue in the spleen trabeculae (Fig. 4f). But the lesions in the spleens from the CIV/Sp group (Fig. 4g) tended to be more pronounced than those in the Sp-only group. The lung of the mice in the CIV-only group revealed widening of the pulmonary interstitial and lung congestion (Fig. 4i). Microscopic lesions of the lung of mice infected with Sp alone typically consisted of multifocal areas of moderate to severe interstitial thickening of alveoli with mononuclear cells (Fig. 4j). The CIV/Sp group showed similar lung congestion but severe fibrin exudation along with accumulations of neutrophils in the bronchi and widened alveolar septa (Fig. 4k).

**Fig. 5:**
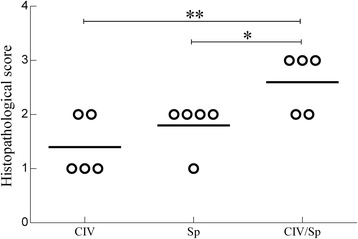
Degree of lung injury present in mice after infection with CIV alone, *S. pseudintermedius* alone, or both. The lungs were assigned a grade from 0 to 3 based on the histological character of the lesions. Analysis of variance (ANOVA) was performed for the statistical analysis. *, *P* < 0.05, or **, *P* < 0.01

The histopathological scores for the H&E sections of the lungs from mice were determined (Fig. 5, Additional file 1). The histopathological score given to the lungs of the mice in the CIV/Sp group was significantly higher than those of the CIV group (*P*<0.01) and the Sp group (*P*<0.05).

### Determination of cytokine concentrations

**Fig. 6:**
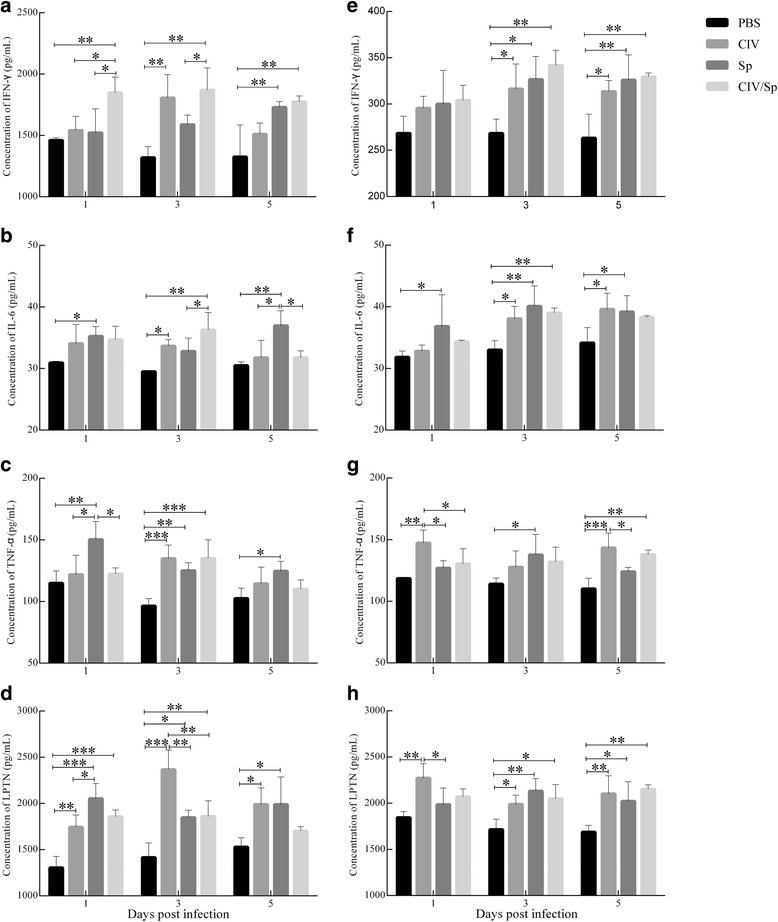
Characterization of IFN-γ (**a**, **e**), IL-6 (**b**, **f**), TNF-α (**c**, **g**) and Lptn (**d**, **f**) secretion from the spleen (**a**, **b**, **c** and **d**) and lung (**a**, **b**, **c** and **d**) tissues of mice after CIV alone, *S. pseudintermedius* alone, or both. The concentrations in the supernatants of homogenates from the spleens and lungs on days 1, 3 and 5 post-challenge were measured by ELISA. The results are expressed in terms of pg/ml. Analysis of variance (ANOVA) was performed for the statistical analysis. *, *P* < 0.05, **, *P* < 0.01, or ***, *P* < 0.001

The levels of IFN**-**γ, TNF-α, IL-6 and Lptn in the spleen and lung of mice at 1, 3 and 5 d.p.i. were measured by ELISA (Fig. 6, Additional file 1). The levels of all four cytokines in the spleen and lung were elevated in the CIV-only, Sp-only and CIV/sp groups compared with the PBS group. The most pronounced difference was found at 3 d.p.i.. There were significant values (*P*<0.01 or *P*<0.05) between the three infection groups and the control group with the exception of the IFN**-**γ and IL-6 levels in the spleen between the Sp-only and PBS group and the TNF-α levels of the lung between the CIV-only or CIV/Sp group and the PBS group.

Compared with the CIV-only and Sp-only groups, at 1, 3 and 5 d.p.i., the levels of IFN-γ in the spleen and lung were higher in the CIV/Sp group, and IFN-γ level of the spleen reached the statistical significance at 1 d.p.i. (*P* < 0.01 or *P* < 0.05). At 1 d.p.i., the IL-6 levels of the spleen and lung showed a significant difference between the Sp and PBS groups. At 5 d.p.i., the IL-6 levels of the spleen in the Sp-only group were significantly higher compared with the levels in the CIV-only and CIV/Sp groups (*P*<0.01 or *P*<0.05), whereas there was no significant difference in the IL-6 levels in the lung between either the Sp-only or CIV-only group and the CIV/Sp group. In the case of TNF-α and Lptn, at 1 and 5 d.p.i., the highest levels in the spleen were observed in the Sp-only group, whereas the highest levels in the lung were found in the CIV-only group. There was no significant difference between the Sp-only or CIV-only group and the CIV/Sp group.

## Discussion

Several studies have reported that influenza virus A is extremely contagious to humans and domestic animals, including avian, swine, equine, canine and feline species [31], and causes significant losses to animals and humans. Infection with influenza virus alone is rarely fatal but can encourage secondary bacterial infections that lead to death [32]. Sp is a common commensal in dogs and can cause opportunistic infections [33]. In this study, the coinfection of CIV and Sp resulted in more severe clinical signs and body weight loss in mice and an increased severity of microscopic lesions in the brain, spleen and lung compared with infection with either pathogen alone. The findings of this study showed that the interaction between the two pathogens appears to generate a more severe outcome than that observed with the individual pathogens by themselves.

Our study demonstrated increased bacterial loads in the blood, brain, spleen and lung of the mice in the CIV/Sp group compared with those in the Sp-only group. The finding is in agreement with observations from an earlier study conducted by Lee et al. [25] in which mice co-infected with virus and bacteria showed a higher bacterial load in the blood, spleen, kidneys and liver at 4 and 72 h after staphylococcal infection compared with the mice infected with bacteria alone. In another study, mice exposed to influenza virus followed by *S. pneumoniae* showed greater bacterial counts after 24 and 48 h of infection [34]. The nature of the interaction between CIV and Sp is unknown. However, studies have shown that influenza A virus infection leads to increased susceptibility of the host to secondary bacterial infection by damaging the respiratory epithelial barrier, upregulating host expression of receptors for bacteria, and impairing the innate immune response [35, 36]. Bacterial clearance can be prevented by viral-induced abnormalities in the immune response [37–39]. Previous data suggest that the increased bacterial titres in the lungs, blood and secondary organs cause high mortality and morbidity in groups of mice co-infected with *S. aureus* 3 and 7 d after influenza virus A infection [29]. However, in the present study, no mortality was noted; this finding may be because CIV H3N2 is not lethal to mice [5, 6].

The present study showed that the bacterial loads in the blood, brain, spleen and lungs peaked in Sp-only and CIV/sp groups on d.p.i. 2, indicating that the bacteria can enter the bloodstream, are transported to the other tissues and then pass through the blood–brain barrier into the brain, thus causing severe neurological signs. Distinct central nervous disturbances observed at this time, including bending of the head towards one side and walking in circles, support this hypothesis. More severe neurologic signs were observed in the coinfection group. Interestingly, in addition to an increased bacterial burden, we found that the viral loads in the blood, brain, spleen and lung were augmented following bacterial infection. Smith et al. [40] used a kinetic model to explore the coupled interactions of influenza A virus and *S. pneumoniae* and reported that the subsequent *S. pneumoniae* infection enhances viral release from infected cells. Therefore, we speculate that the observed increase in viral loads after bacterial infection may be due to increased virus release via bacterial proteases. Additionally, we cannot rule out the possible mechanism that elevated viral loads in the presence of bacteria are due to a reduction in viral clearance. Further work is necessary to study the precise mechanism involved.

The loads of both CIV and Sp in the coinfected mice were greater compared with those of singly infected mice. These results may help explain the increased severity of the lesions observed in the coinfected mice. Additionally, cytokine production after infection likely plays a role in the pathological changes observed during infection. A previous study reported that an excessive production of TNF-α leads to immunopathology [41]. The study conducted by Lee et al. [42] reported that the levels of IFN-γ and TNF-α in the lungs of dogs infected with H3N2 influenza virus increased rapidly, and the infected dogs developed severe bronchointerstitial pneumonia accompanied by a massive infiltration of immune cells. Lymphotactin is a chemokine that recruits T and NK cells, and can regulate or modulate T cell–mediated immune responses [43]. A previous report showed mycobacterial antigen could induce an increase of Lptn transcript, while Lptn has a regulation role in IFN-γ production [44]. A study from van Berke [45] reported that a gamma herpesvirus selective chemokine binding protein could inhibit Lptn action. In the present study, we determined the concentrations of IFN-γ, IL-6, TNF-α and Lptn in the lungs and spleen of mice infected with Sp alone, CIV alone or both CIV and Sp. In general, higher levels of cytokines were observed in all of the infection groups compared with the uninfected animals. We speculated that elevated levels of cytokines may be associated with the clinical manifestations observed in the infected mice. The comparison among the three infection groups revealed that IFN-γ showed the largest changes in the coinfected mice compared with the mice infected with either agent alone, particularly on day 1. These data are consistent with previous reports in which a higher level of IFN-γ expression was observed after coinfection with influenza virus and bacteria [25, 46]. IFN-γ is a Th1 cytokine that can activate macrophages to produce nitric oxide and other inflammatory mediators [47]. This finding may explain the increased severity of lesions observed early in the coinfected mice compared with the mice infected with bacteria or virus alone. With the exception of IFN-γ, the other cytokine or chemokine levels observed in coinfected mice showed no significant differences from those observed in mice infected with CIV or Sp alone. These data showed that coinfection did not influence the production levels of IL-6, TNF-α and Lptn.

Although mice are extensively used for influenza research, and they can be successfully infected with CIV [5, 6], the use of mouse model can be limited, however, due to the fact that mice are not natural hosts for CIV. The signs including lethargy, anorexia and body weight loss, huddling and ruffled fur, which are characteristic of influenza virus infection in mice [48], could be seen. However, some signs in dog disease such as coughing, nasal discharge and fever have not been observed. In the future study, we will further perform coinfection of CIV and Sp in beagles.

## Conclusions

Taken together, the findings presented in this study showed that coinfection with CIV and Sp may worsen clinical signs and lesions in mice. This additive effect supports the need for improving the current strategies for the control of influenza and secondary bacterial infection. Because CIV is a relatively recently described canine pathogen, the available clinical data are relatively limited, and further epidemiological studies on the interactions between this virus and secondary bacterial infection are required.

## Abbreviations

ANOVA, Analysis of variance; CFU, Colony forming unit; CIV, Canine influenza virus; ELISA, Enzyme Linked Immunosorbent Assay; IFN-γ, Interferon-gamma; IL-6, Interleukin 6; LPTN, Lymphotactin; MDCK, Madin-Darby canine kidney; PBS, phosphate-buffered saline; PCR, Polymerized chain reaction; PFU, Plague forming unit; RNA, Ribonucleic acid; SP, *Staphylococcus pseudintermedius*; TNF-α, Tumor necrosis factor.

## Additional file

Additional file 1: Availability of data and materials of the reserch. (PDF 94 kb)
