# Supplementary material for: Canine influenza virus coinfection with Staphylococcus pseudintermedius enhances bacterial colonization, virus load and clinical presentation in mice
Source: BMC Vet Res. 2016 Jun 4;12:87. doi: 10.1186/s12917-016-0708-6 (PMC4893303; doi:10.1186/s12917-016-0708-6)
Supplement: Additional file 1: — Availability of data and materials of the reserch. (PDF 94 kb) [file 12917_2016_708_MOESM1_ESM.pdf]

Fig. 1Changes in the body weights of BALB/c mice that were infected with CIV or *S. pseudintermedius* alone, coinfected with both, or exposed to PBS as a control.

	PBS		CIV		Sp		CIV/Sp	
day	mean	sd	mean	sd	mean	sd	mean	sd
0d	100.00	0.00	100.00	0.00	100.00	0.00	100.00	0.00
1d	103.86	1.82	100.80	1.19	93.22	3.18	92.48	3.97
2d	103.96	1.66	101.88	2.29	91.82	4.90	89.86	3.33
3d	104.65	1.22	101.10	2.48	92.05	3.64	91.01	3.12
4d	107.52	1.33	100.37	2.97	94.13	2.59	90.77	4.83
5d	109.51	2.10	102.14	2.57	94.48	2.63	90.71	4.59

Fig. 2. **Dynamic change in the bacteria loads in the blood, brain, spleen and lung of mice infected with *S. pseudintermedius* alone or both *S. pseudintermedius* and CIV**.

blood

day	Sp			CIV/Sp		
1d	3.00	2.90	3.70	3.15	3.75	3.20
2d	3.30	2.90	3.10	4.15	5.00	4.00
3d	2.70	2.60	2.30	2.60	2.70	3.34
4d	2.30	2.70	2.30	2.70	2.60	2.30
5d	2.60	2.30	2.18	2.48	2.30	2.85

brain

day	Sp			CIV/Sp		
1d	2.82	2.82	2.52	4.00	2.70	2.61
2d	4.13	4.51	4.62	4.62	5.72	4.61
3d	4.53	3.58	4.86	4.99	4.89	4.76
4d	4.85	4.74	5.10	5.29	4.89	5.19
5d	3.99	4.95	5.18	4.64	5.71	4.66

spleen

day	Sp			CIV/Sp		
1d	3.21	3.30	3.32	4.59	4.34	4.31
2d	5.25	5.20	5.75	6.51	6.38	6.45
3d	4.36	4.71	4.53	5.43	5.41	5.42
4d	3.59	4.42	4.92	4.91	5.29	4.14
5d	3.53	4.23	4.37	4.18	4.12	4.59

lung

day	Sp			CIV/Sp		
1d	2.99	3.88	3.69	4.53	3.62	3.80
2d	5.53	5.40	5.51	6.31	5.51	5.91
3d	4.09	4.34	4.46	4.54	5.32	4.11
4d	4.36	4.49	4.24	4.32	4.39	4.64
5d	4.15	3.98	4.44	4.26	4.26	4.06

Fig. 3. Dynamic change in the viral loads in the blood, brain, spleen and lung of mice infected with CIV alone, or both *S. pseudintermedius* and CIV.

blood

day	CIV			CIV/Sp		
1d	3.58	3.67	3.80	3.73	3.84	3.63
2d	3.49	3.44	3.44	3.49	3.49	3.54
3d	3.47	3.50	3.38	3.65	3.57	3.64
4d	3.58	3.63	3.58	3.92	3.83	3.83
5d	3.57	3.60	3.61	3.65	3.64	3.64

brain

day	CIV			CIV/Sp		
1d	3.53	3.55	3.55	3.72	3.80	3.72
2d	3.18	3.21	3.21	3.65	3.64	3.55
3d	3.37	3.45	3.39	3.51	3.67	3.66
4d	3.33	3.03	3.20	3.63	3.44	3.63
5d	3.12	3.30	3.24	3.34	3.59	3.58

spleen

day	CIV			CIV/Sp		
1d	3.58	3.64	3.51	3.68	3.53	3.66
2d	2.66	3.39	3.36	3.63	3.65	3.58
3d	3.40	3.42	3.36	3.29	3.71	3.41
4d	3.32	3.17	3.09	3.49	3.51	3.49
5d	3.46	3.02	3.23	3.53	3.51	3.54

lung

day	CIV			CIV/Sp		
1d	3.66	3.67	3.63	3.68	3.72	3.66
2d	3.14	3.18	3.34	3.62	3.67	3.68
3d	3.43	3.68	3.22	3.38	3.61	3.67
4d	3.75	3.01	3.42	3.53	3.61	3.58
5d	3.22	3.51	3.14	3.30	3.29	3.54

Fig. 5. Degree of lung injury present in mice after infection with CIV alone, *S. pseudintermedius* alone, or both.

Number	CIV	Sp	CIV/Sp
1	1	2	3
2	1	2	3
3	1	2	3
4	2	1	2
5	2	2	2

Fig. 6. Characterization of IFN-γ, IL-6, TNF-α and Lptn secretion from the spleen and lung tissues of mice after CIV alone, *S. pseudintermedius* alone, or both.

IFN-γ spleen

day	PBS			CIV			Sp			CIV/Sp		
1d	1478	1437	1468	1669	1507	1448	1386	1745	1436	1910	1702	1932
3d	1422	1281	1257	2019	1657	1745	1580	1513	1669	2069	1826	1717
5d	1557	1375	1045	1437	1610	1485	1708	1783	1697	1736	1761	1828

IFN-γ lung

day	PBS			CIV			Sp			CIV/Sp		
1d	259.3	289.8	255.9	292.7	284.3	309.6	340.1	291.0	269.0	321.5	301.2	289.3
3d	250.9	279.6	274.5	346.8	296.1	306.2	340.1	297.8	341.8	353.6	348.5	323.2
5d	272.9	234.0	283.0	301.2	314.7	324.8	357.0	314.7	306.2	329.9	324.8	333.3

IL-6 spleen

day	PBS			CIV			Sp			CIV/Sp		
1d	30.65	31.17	30.99	31.77	32.80	37.59	36.39	35.88	33.48	32.28	36.56	35.19
3d	29.45	29.45	29.62	33.14	34.85	32.97	30.74	32.62	35.02	34.17	39.47	35.19
5d	30.99	30.65	29.80	29.88	30.40	35.02	34.17	38.45	38.28	31.60	32.97	30.74

IL-6 lung

day	PBS			CIV			Sp			CIV/Sp		
1d	30.83	32.83	31.83	33.82	31.82	32.82	40.86	38.60	31.06	34.08	34.58	34.33
3d	31.58	32.83	34.59	40.36	37.34	36.59	37.84	38.60	43.87	39.60	39.35	38.10
5d	35.60	31.33	35.60	36.59	41.11	41.11	41.86	36.59	39.10	38.10	38.10	38.60

TNF-α spleen

day	PBS			CIV			Sp			CIV/Sp		
1d	124.2	104.5	116.0	137.9	121.4	106.7	149.9	136.0	165.2	126.4	116.9	123.9
3d	93.7	103.3	92.5	123.3	144.9	136.6	123.3	120.1	132.2	135.3	120.1	149.9
5d	110.2	103.9	93.7	128.3	101.7	113.7	128.3	130.2	115.6	115.6	113.1	101.7

TNF-α lung

day	PBS			CIV			Sp			CIV/Sp		
1d	119.0	118.9	117.7	140.9	141.5	159.4	131.9	120.4	128.7	119.1	128.7	143.4
3d	111.9	110.6	119.6	142.8	122.3	118.5	127.4	128.7	156.9	135.1	119.1	142.2
5d	114.5	100.4	115.7	134.5	138.9	156.9	122.3	128.1	121.7	140.9	133.8	138.9

Lptn spleen

day	PBS			CIV			Sp			CIV/Sp		
1d	1188	1434	1292	1710	1889	1634	2078	2201	1880	1908	1889	1776
3d	1387	1585	1273	2210	2286	2607	1899	1757	1889	2050	1804	1729
5d	1604	1566	1415	2173	1984	1814	2012	2277	1681	1672	1672	1757

Lptn lung

day	PBS			CIV			Sp			CIV/Sp		
1d	1818	1798	1919	2209	2159	2449	1918	1848	2189	1968	2129	2109
3d	1618	1688	1838	1958	1908	2098	2149	1988	2259	2088	2179	1878
5d	1708	1608	1748	2048	2319	1938	2229	1808	2028	2098	2189	2169
